# An Operational In Situ Soil Moisture & Soil Temperature Monitoring Network for West Wales, UK: The WSMN Network

**DOI:** 10.3390/s17071481

**Published:** 2017-06-23

**Authors:** George P. Petropoulos, Jon P. McCalmont

**Affiliations:** 1 Department of Geography and Earth Sciences, University of Aberystwyth, Aberystwyth SY23 3DB, UK; 2 Institute of Biological Environmental and Rural Sciences, University of Aberystwyth, Aberystwyth SY23 3EE, UK; jpm8@aber.ac.uk

**Keywords:** soil moisture, soil temperature, in situ, monitoring networks, WSMN, Wales

## Abstract

This paper describes a soil moisture dataset that has been collecting ground measurements of soil moisture, soil temperature and related parameters for west Wales, United Kingdom. Already acquired in situ data have been archived to the autonomous Wales Soil Moisture Network (WSMN) since its foundation in July 2011. The sites from which measurements are being collected represent a range of conditions typical of the Welsh environment, with climate ranging from oceanic to temperate and a range of the most typical land use/cover types found in Wales. At present, WSMN consists of a total of nine monitoring sites across the area with a concentration of sites in three sub-areas around the region of Aberystwyth located in Mid-Wales. The dataset of composed of 0–5 (or 0–10) cm soil moisture, soil temperature, precipitation, and other ancillary data. WSMN data are provided openly to the public via the International Soil Moisture Network (ISMN) platform. At present, WSMN is also rapidly expanding thanks to funding obtained recently which allows more monitoring sites to be added to the network to the wider community interested in using its data.

## 1. Introduction

Land surface interactions govern critical exchanges of energy and mass between the terrestrial biosphere and the atmosphere, and are major drivers of the Earth’s climate system ([1,2]). Today, particularly so in the face of climate change and the need to meet global food and water security requirements, there is an urgent need for a better understanding of natural processes in the Earth system and of land surface interactions (LSIs) [3]. This is now widely recognised by the global scientific community as a matter requiring urgent attention for further investigation [4,5]. In this regard, exact information on the spatiotemporal variation of soil parameters such as soil surface moisture (SSM) and soil temperature (ST) are of key significance due to the specific influence of these parameters on various physical processes of the Earth system, where they exert a strong control on the Earth’s water cycle and ecosystem functioning in general [6,7]. For example, SSM controls the partitioning of available energy at the ground surface into latent (LE) and sensible (H) heat exchange through evaporation and transpiration processes, thus linking the water and energy balances through the moisture and temperature states of the soil [8]. Water, whether contained in the air or the soil, has specific heat capacity which is orders of magnitude greater than dry air and any site which displays a large variation in soil water content across time will equally display such variation in its capacity to absorb, store, and release heat energy [9]. Thus, accurate information on the variation of these parameters over both time and space domains is imperative for a number of environmental and commercial applications, from sustainable water resource management to evaluating parameterization schemes for weather and climatic models [10].

There are many different options that can be considered for deriving both SSM and ST based on the use of ground instrumentation, each having distinct practical advantages and disadvantages (e.g., see recent reviews by [11,12]). Generally, the use of ground instrumentation has certain advantages, such as a relatively direct measurement; instrument portability; easy installation, operation and maintenance; the ability to provide measurement at different depths; and also the relative maturity of the methods. Nevertheless, ground measurement techniques have proven very difficult to implement practically over large areas. This is mainly because they can be complex, expensive, labour-intensive, and often intrusive at the study site where are installed. In addition, their use often requires the deployment of extensive equipment in the field in order to provide only localised estimates of SSM, making them unsuitable for measuring this parameter over large spatial scales.

In view of the importance of information on the spatial distribution of SSM, various operational ground-based global observational networks have been developed over the last decade or so, providing SSM/ST at no cost (see [12] for a review of existing networks). These networks aim to systematically collect, archive, and openly distribute to the users’ community a wide variety of such data acquired under different ecosystem and topographical conditions around the globe. Nowadays, it has been recognised as being of pivotal importance the establishment and maintenance of such networks [7]. Indeed, data from such “operational” networks are crucial for advancing our understanding of the physical processes involved in water and energy exchanges at local scale [12]. Also, they are important for performing multi-scale analyses exploiting either land surface models or remote sensing data, for example in validating modelling predictions or remote sensing estimates [13]. Furthermore, such data is also crucial for the benchmarking of Earth Observation-based relevant algorithms or operational products, where ground measurements are often used as the “reference” dataset against which the model predictions are compared. Conducting such studies is a key step towards the development of new retrieval algorithms as well as for the distribution of operational products before they are made available for use in the wider community [14,15]. Thus, a continuation of the initiatives supporting and expanding such ground observational networks in the future is a very valuable investment from multiple perspectives, and its importance cannot be overstated.

While there are now several long-term data sets of SSM and ST available worldwide provided via platforms such as the International Soil Moisture Network (ISMN, [13]), to our knowledge, there are very few such sites set up in the United Kingdom. For Mid-Wales a network helping to understand hydrology and soil water storage may be particularly useful for an area that supplies several major English cities with their drinking water and, sadly, a large proportion of their flood water. Such freely available information could be extremely useful for a wide range of purposes, such as livestock protection, yield prediction, flood forecasting, and human health. This paper provides a description of a 5+ year (and ongoing) Wales Soil Moisture Network (WSMN) dataset produced from ground instrumentation installed in the vicinity of Aberystwyth, Wales, UK.

## 2. Overview

An operational in situ monitoring network of soil moisture and temperature monitoring has been established in Wales, UK. WSMN has been functioning as a data collection network since 2011 and provides measurements, in near real time and on a long term basis, of SSM, ST, precipitation, and incoming solar radiation (Rg) at regular intervals of either 30 min or an hour, varying by site. More information about WSMN can be found at the network web site as well (http://www.aber.ac.uk/wsmn). As well as two dedication stations, this network currently incorporates monitoring stations installed as part of other, wider research projects (Carbo-Biocrop, ELUM, BSBEC, and the Pwll Peiran Upland Research Platform).

**Figure 1:**
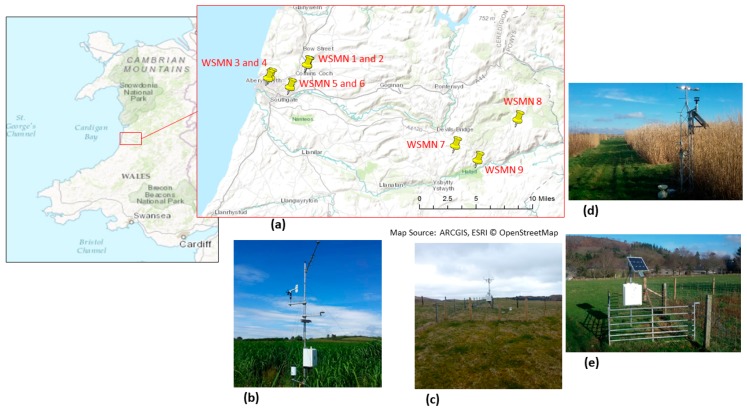
(**a**) Overview of the locations on which the WSMN experimental sites are installed to acquire SM, ST, and ancillary data. Examples of images of the sites from: (**b**) Penglais, (**c**) Pwllpeiran, (**d**) Cae-Canol (**e**) Comins-Coch.

Briefly, WSMN currently consists of nine stations spread across five sites (see Figure 1) situated in the Aberystwyth region, West Wales, UK.

Sites 1 and 2 are located in an agricultural grassland site around three miles east of Aberystwyth, near to the Gogerddan campus of Aberystwyth University. Sites 3 and 4 are located on a six hectare site of bioenergy crops (*Miscanthus x giganteus*) at the northern outskirts of Aberystwyth. Sites 5 and 6 are located on the eastern edge of Aberystwyth, near to the Llanbadarn campus of Aberystwyth University, these sites are part of a separate trial of *Miscanthus* and short-rotation coppice willow plots and as such the field has many individual plots across it. Site 5 has sensors located under one of the Miscanthus plots, site 6 under the willow. Sites 7, 8, and 9 are located under grassland on the Pwllpeiran Research Farm, near Devil’s Bridges, around 17 km east of Aberystwyth. These sites are at much higher altitudes compared to the other six sites and as such provide a good opportunity for comparison between highlands and lowlands in the Aberystwyth area. For further details about each site see Table 1 below. Site 7 is located under upland, grazed grassland while site 8 is at higher altitude under semi-natural, peat grassland. Site 9 was recently added, June 2016, close to the Pwll Peiran research farm to trial a new set of sensors (SM-150, Delta-T devices, Cambridge, UK) and to extend the altitude gradient across the Pwll Peiran estate. Some of the soil moisture sensors and accompanying monitoring have been established specifically under Miscanthus under a research project which aimed at understanding the environmental implications of land-use change to these bioenergy crops. There are currently 7000 hectares of Miscanthus being grown in the UK supplying newly opened, dedicated straw burning power plants. With the ending of the CAP, farmers are beginning to look for new markets and opportunities, developing domestic biomass markets, both large and small scale, is likely to be a key move for agriculture, particularly in marginal economic areas such as Wales where farm viability is currently completely dependent on subsidy payments.

## 3. Scientific Importance and Use of Data

The data acquired from WSMN can be used for a wide range of purposes. First of all, the availability of this new in situ dataset provides a unique contribution towards the development at different scales of long-term monitoring capability of land-surface interactions for Atlantic environments such as that of west Wales. Indeed, access to such data is of pivotal value to the region as it is expected to aid advancing our understanding of the physical processes involved in water and energy exchanges first of all at a local scale. Moreover, in larger scale studies such data can be incorporated into wider networks and used as forcing data in the development and validation of land surface process models (e.g., see [3,16,17]) or can be integrated with satellite data (e.g., via data assimilation schemes, e.g., [18,19]). WSMN is capable of providing data for studies focusing on spatio-temporal scaling of SSM & ST. Indeed, as both parameters can vary considerably in time as well as space, it is clear that the high temporal resolution of the WSMN dataset can play a very useful role in understanding and developing methods to improve our understanding of this spatiotemporal variability. The WSMN data is also well-suited to be used in studies aimed at evaluating the capability of different EO-based algorithms and sensing systems in deriving SSM or ST (e.g., [14,15]). Indeed, taking into account the increasing number of satellite missions being placed in orbit today capable of providing estimates of SSM and ST has made it indispensable to provide “reference” data across a wide range of environments and climates globally. In this context, at a broader scale, this study will assist towards an objective evaluation of the retrieval accuracy of relevant satellite-derived SSM/ST estimations for the region of Wales, something that to our knowledge is at present largely lacking or under-developed. However, it should be noted that some of the WSMN sites are in close proximity to the sea and those may not be so suitable to be used for validating specifically coarse resolution SSM products.

Furthermore, in addition to the existing nine stations (eight datasets currently available on the ISMN website with the ninth station to be added), there are plans (and funding in place) to extend this to around 20 sites across a wider area of Mid-Wales. This will provide a much wider picture of soil moisture and extend the altitudinal gradient which is key to understanding hydrological dynamics, both in terms of flood risk and drought buffering. At the same time, some of the available funding will be spent to complement the existing stations (i.e., install a sensor at 5 cm depth at sites 5–8 and at 10 cm depth at sites 1–4 and 9), which will allow a more consistent dataset.

## 4. Data Summary

### 4.1. Soil Moisture

**Figure 2:**
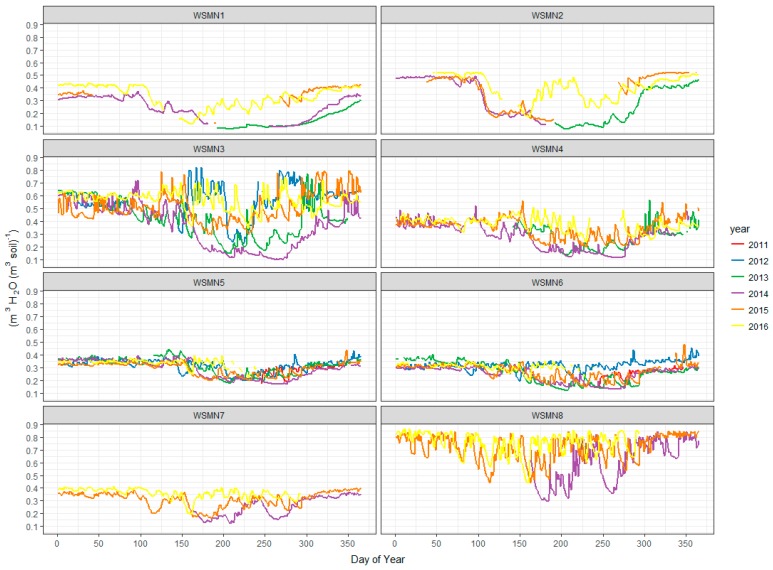
Time series of SSM at 5 cm depth from the soil surface layer measured at the WSMN experimental sites from their individual installation dates to the present.

Soil moisture measurements are acquired across the sites at a range of logged intervals (15, 30, and 60 min, see Table 1), see Figure 2 for a plot of data recorded to date at each site. Information on the soil texture and bulk density for each site will be provided on the WSMN web site. For all sites, time domain reflectometry (TDR) instrumentation is used for the measurement of SSM. TDR is a method that uses propagation of a high-frequency transverse electromagnetic wave along a cable attached to a parallel conducting probe inserted into the soil. The signal is reflected from one probe to the other before being returned to the meter that measures the time elapsed between sending the pulse and receiving the reflected wave. Assuming that the cable and waveguide length are known, the propagation velocity, which is inversely proportional to the dielectric constant, can be directly related (through calibration) to SSM. This provides a measurement of the average volumetric water content in a soil volume along the length of the waveguide [7,11]. In the WSMN sites TDR probes have been permanently installed in each site in the ground horizontally at ~5–10 cm depth from the soil surface layer. The recorded ST (see next section) is used to correct the soil moisture probes. All soil moisture data is 0.1% *v*/*v* resolution with a range of 0–50% *v*/*v* at all sites. All the sensors installed are well within the factory calibration that the company providing the data (i.e., Campbell) supply.

### 4.2. Soil Temperature

**Figure 3:**
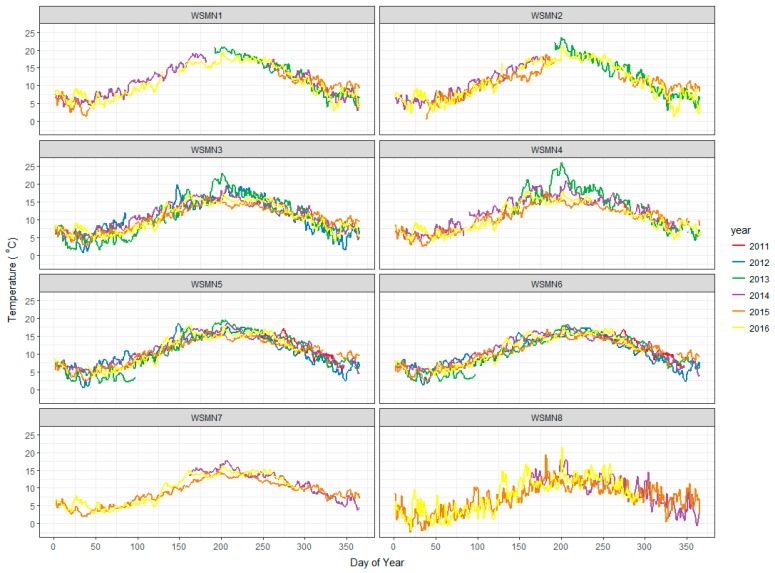
Time series of ST at 5 cm depth from the soil surface layer measured at the WSMN experimental sites from their individual installation dates to the present.

Another parameter that has been measured systematically at all sites is ST, which is also logged as a mean for each site specific recording interval (see Table 1) at the same depth where the soil moisture probes have been installed (i.e., ~5–10 cm below soil surface layer). Soil temperature data are required for temperature correcting the soil moisture sensors, which is built into the data logging programs. Figure 3 below summarises the ST measurements collected since the network was set up in 2011, derived from the daily averaging of the ST values. As can be seen, changes in ST follow the seasonal patterns at all sites with peaks occurring during summertime and lowest values observed during winter. Inter-annual variation is notable at all sites and is typically within a range of about 5 °C, the largest inter-annual variation can be seen at the highest altitude site (WSMN8) which also shows a wider day to day variation, likely driven by the harsher climate and highly organic soil. 

### 4.3. Ancillary Data

**Figure 4:**
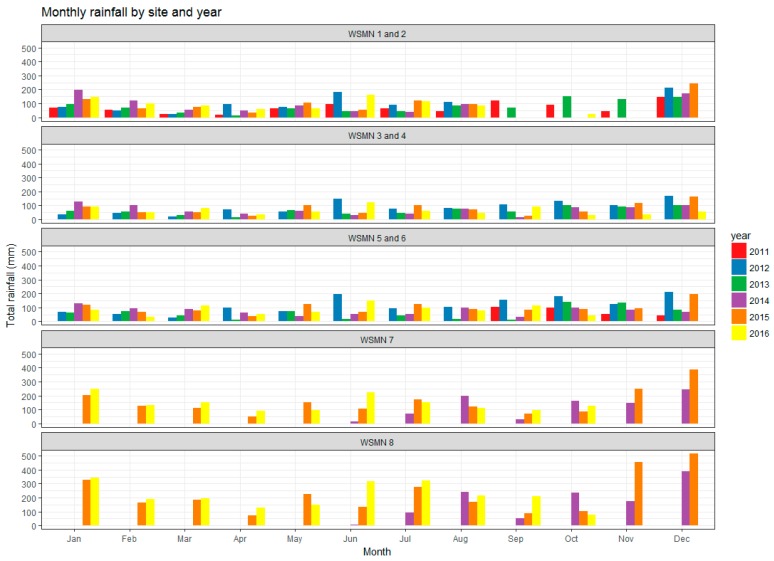
Monthly totals of rainfall measurements from the WSMN sites collected between 2011 and 2016.

Cumulative rainfall is collected specifically at all sites at each individual site logging interval (15, 30, or 60 min) except at site 7 where, to date, only daily total rainfall is recorded. Sites 3 and 4 do not have their own dedicated rain gauge but are adjacent to the UK Met Office Gogerddan station where daily rainfall is available (http://www.metoffice.gov.uk). Rainfall is recorded by tipping bucket rain gauges (Young’s 52203-Sites 3–4, ARG100-Sites 5–6 and 7–8. CSI, Logan, UT, USA). Collected rainfall data is made available without the implementation of any pre-processing (e.g., for undercatch using different shelter correction factors depending on the exposure of a site to wind). Figure 4 below shows the total monthly rainfall acquired by site and year since WSMN was set up in 2011 until end of 2016. Of note is the large difference in the rainfall amounts reported between the lowland (sites 1–6) and upland sites (sites 7–8) with the rainfall in the uplands being significantly higher. Seasonally, similar trends are observed in terms of precipitation amounts between the sites 1–6 as a group and also separately for sites 7–8 as another group. This is mainly attributed to the difference in the elevation on which those groups of sites are located.

## 5. Data Quality 

At present, the WSMN data is collected on a weekly to monthly basis with collection carried out manually during routine research fieldwork being carried out at the sites. However, in future data collections from the expanding network—which is in progress—will become automated through GPRS communication sensors into a central database for ongoing quality control, harmonization, and distribution to the wider ISMN database. Following the data acquisition, collected data from the monitoring sites are visually inspected to identify any erroneous errors (e.g., anomalous and non-physical values). This step also includes comparisons between SSM with ST and compared to rainfall and incoming solar radiation where available. This checking is a standard process done in other similar operational networks (e.g., [19]). All detected erroneous data are then removed from the database and flagged as missing. No gap filling of data has been undertaken, with all missing or poor quality data being flagged as ‘N/A’. Within a process that is becoming at present fully automated, collected data will be harmonised in terms of measurement unit, sampling interval, and metadata, and after a basic quality check stored in a database. An operational algorithm developed in-house is used to generate a list of files that summarise the values, including hourly, daily mean, and monthly mean values for all of the measured variables of each station. The final dataset per site is saved in ASCII and .csv (spreadsheet) formats and is then transferred to the ISMN team and is ready for distribution to potential users’ community for use in further experiments and applications.

## 6. Data Management & Availability 

Data from stations 1 to 8 are currently available via the International Soil Moisture Network (ISMN, [13], https://ismn.geo.tuwien.ac.at/), with data from station 9 to be added in the near future. ISMN is an international cooperation to establish and maintain a global in situ soil moisture database. This international initiative is coordinated by the Global Energy and Water Exchanges Project (GEWEX) in cooperation with the Group of Earth Observation (GEO) and the Committee on Earth Observation Satellites (CEOS). One of the key advantages of ISMN is the fact that it provides easy and rapid access to harmonised and quality-checked SSM ground measurements from a large number of locations distributed all over the world, amalgamating the efforts of different groups attempting to provide long-term measurements of SSM. ISMN, therefore, acts as a hosting and harmonisation facility with the key objective to collect and fuse everything that is available [7].

**Figure 5:**
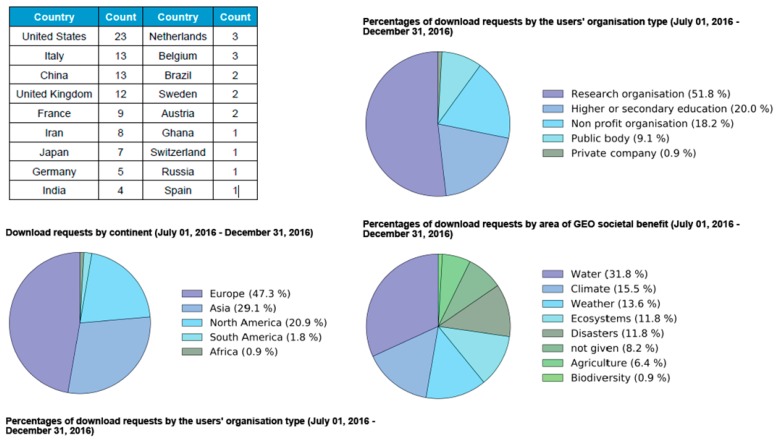
Summarised statistics of the WSMN data usage for the period 1 July 2016 to December 2016.

On the ISMN web site, data viewing is possible without registration, however, to acquire the data one needs first to register, a process that is fast and straightforward. The website provides all of the information required for interpretation of these data (including metadata), along with site photographs, maps, and descriptions. Due acknowledgment in any publication or presentation arising from use of these data is required. WSMN data collection is ongoing as of July 2011 for all WSMN sites, even though data from year 2009 is available for a small number of sites. The data base generated by WSMN is freely available for any research or scientific purpose or practical applications. So far, according to the ISMN download statistics, since the network set up in 2011 and until December 2016 data had been placed in total 366 requests for WSMN data download, which evidences a very good level of data dissemination already. An example of the WSMN data usage for the period 1 July 2016–31 December 2016 is shown in Figure 5.

## 7. Conclusions and Summary

This paper presented an overview of an operational soil surface moisture (SSM) and soil temperature (ST) monitoring network which has been set up in west Wales, UK. The network, named as the “Wales Soil Moisture Network (WSMN)”, has been acquiring and openly distributing data from 5+ years from a total of nine sites (with large scale expansion in process), representative of typical Welsh environments and land use/cover types. All the WSMN data are harmonised in terms of measurement unit, sampling interval (15 min, half-hourly, and hourly means), and metadata and are provided, quality checked, and filtered for spurious observations and are provided openly to the interested community via the ISMN data distribution platform. WSMN has been running operationally on a long-term basis and aims to support Earth Observation, land modelling, and Earth system process studies for oceanic climates such as those found in Wales. More data are expected to become available in the near future thanks to significant funding that was recently secured to facilitate this purpose. Users in Wales also systematically collecting SSM/ST are also welcomed to join WSMN and share their data publicly via the routes already established for this scope. WSMN is an open database and more information about the network can be found at http://www.aber.ac.uk/wsmn whereas direct access to the data can be obtained from ISMN upon registration (https://ismn.geo.tuwien.ac.at/).

## Figures and Tables

**Table 1:** Summary of the main characteristics of the sites belonging to WSMN.

Site	Established	Coordinates	Elevation	Sensor Depth	Land Use
Site 1—Comins Coch (Station 1)	2013	52°25′56.38′′ N 4°1′14.15′′ W	30 m	5 cm	Agriculture/Grasslands
Site 2—Comins Coch (Station 2)	2013	52°25′56.78′′ N 4°1′17.76′′ W	28 m	5 cm	Agriculture/Grasslands
Site 3—Penglais (Station 3)	2012	52°25′20.76′′ N 4°4′6.01′′ W	114 m	5 cm	Ryegrass Pastureland transitions to Miscanthus x Giganteus Bioenergy Crop
Site 4—Penglais (Station 4)	2013	52°25’17.24” N 4°4’14.15” W	110 m	5 cm	Ryegrass Pastureland transitions to *Miscanthus* Bioenergy Crop
Site 5—Cae Canol (Station 5)	2009	52°24′49.99′′ N 4°2′34.42′′ W	128 m	10 cm	16 individual plots each of willow and *Miscanthus* and grass rows
Site 6—Cae Canol (Station 6)	2009	52°24′49.67′′ N 4°2′35.18′′ W	128 m	10 cm	16 individual plots each of willow and *Miscanthus* and grass rows
Site 7—Pwllpeiran (Station 7)	2014	52°21′55.07′′ W 3°49′54.28′′ W	375 m	10 cm	Semi-Improved U4 Grassland
Site 8 –Pwllpeiran (Station 8)	2014	52°23′13.01′′ N 3°45′7.35′′ W	500 m	10 cm	Peatland
Station 9—PwllPeiran (Station 9)	2016	52°21′11.57′′ N 3°48′11.22′′ W	260 m	5 cm	Semi-Improved grassland

## References

[1] Jung M., Reichstein M., Margolis H.A., Cescatti A., Richardson A.D., Arain M.A., Williams C. (2011). Global patterns of land-atmosphere fluxes of carbon dioxide, latent heat, and sensible heat derived from eddy covariance, satellite, and meteorological observations. J. Geophys. Res. Biogeosci..

[2] Srivastava P.K., Han D., Islam T., Petropoulos G.P., Gupta M., Dai Q. (2016). Seasonal evaluation of Evapotranspiration fluxes from MODIS Satellite and Mesoscale Model Downscaled Global Reanalysis Datasets. Theor. Appl. Climatol..

[3] Ireland G., Petropoulos G.P., Carlson T.N., Purdy S. (2015). Addressing the ability of a land biosphere model to predict key biophysical vegetation characterisation parameters with Global Sensitivity Analysis. Environ. Model. Softw..

[4] 2000/60/EC of the European Parliament and of the Council of 23 October 2000 Establishing a Framework for Community Action in the Field of Water Policy. http://eur-lex.europa.eu/resource.html?uri=cellar:5c835afb-2ec6-4577-bdf8-756d3d694eeb.0004.02/DOC_1&format=pdf.

[5] Stocker T.F., Qin D., Plattner G.-K., Tignor M., Allen S.K., Boschung J., Nauels A., Xia Y., Bex V., Midgley P.M., IPCC (2013). Climate Change. The Physical Science Basis. http://www.ipcc.ch/report/ar5/wg1/.

[6] Shen C., Niu J., Phanikumar M.S. (2013). Evaluating controls on coupled hydrologic and vegetation dynamics in a humid continental climate watershed using a subsurface-land surface processes model. Water Resour. Res..

[7] Petropoulos G.P., Ireland G., Barrett B. (2015). Surface Soil Moisture Retrievals from Remote Sensing: Evolution, Current Status, Products & Future Trends. Phys. Chem. Earth.

[8] Vereecken H., Huisman J.A., Pachepsky Y., Montzka C., van der Kruk J., Bogena H., Weihermüllera L., Herbsta M., Martinez G., Vanderborght J. (2013). On the spatio-temporal dynamics of soil moisture at the field scale. J. Hydrol..

[9] Oke T.R. (1992). Boundary Layer Climates.

[10] Liu J.G., Xie Z.H. (2013). Improving simulation of soil moisture in China using a multiple meteorological forcing ensemble approach. Hydrol. Earth Syst. Sci. Dis..

[11] Verstraeten W.W., Veroustraete F., Feyen J. (2013). Assessment of evapotranspiration and soil moisture content across different scales of observation. Sensors.

[12] Petropoulos G., Griffiths H., Dorigo W. (2013). Surface Soil Moisture Estimation: Principles and Conventional Measurement Techniques. Remote Sensing of Land Surface Turbulent Fluxes & Soil Moisture.

[13] Dorigo W.A., Wagner W., Hohensinn R., Hahn S., Paulik C., Drusch M., Mecklenburg S., van Oevelen P., Robock A., Jackson T. (2011). The international soil moisture network: A data hosting facility for global in situ soil moisture measurements. Hydrol. Earth Syst. Sci..

[14] Petropoulos G.P., Ireland G., Srivastava P.K., Ioannou-Katidis P. (2014). An appraisal of soil moisture operational estimates accuracy from SMOS MIRAS using validated in-situ observations acquired at a Mediterranean environment. Int. J. Remote Sens..

[15] Petropoulos G.P., Ireland G., Lamine S., Ghilain N., Anagnostopoulos V., North M.R., Srivastava P.K., Georgopoulou H. (2016). Evapotranspiration Estimates from SEVIRI to Support Sustainable Water Management. J. Appl. Earth Obs. Geoinf..

[16] Richter H., Western A., Chiew F. (2004). The effect of soil and vegetation parameters in the ECMWF land surface scheme. J. Hydrometeorol..

[17] North M.R., Petropoulos G.P., Rentall D.V., Ireland G.I., McCalmont J.P. (2015). Quantifying the prediction accuracy of a 1-D SVAT model at a range of ecosystems in the USA and Australia: Evidence towards its use as a tool to study Earth’s system interactions. Earth Surf. Dyn. Discuss..

[18] Petropoulos G.P., Carlson T.N. (2011). Retrievals of turbulent heat fluxes and soil moisture content by Remote Sensing. Advances in Environmental Remote Sensing: Sensors, Algorithms, and Applications.

[19] Smith A.B., Walker J.P., Western A.W., Young R.I., Ellett K.M., Pipunic R.C., Grayson R.B., Siriwardena L., Chiew F.H.S., Richter H. (2012). The Murrumbidgee soil moisture monitoring network data set. Water Resour. Res..

